# Meeting the demand of women affected by ongoing crisis: Increasing contraceptive prevalence in North and South Kivu, Democratic Republic of the Congo

**DOI:** 10.1371/journal.pone.0219990

**Published:** 2019-07-19

**Authors:** Sara E. Casey, Meghan C. Gallagher, Erin Files Dumas, Jessica Kakesa, Justin Mumbere Katsongo, Jean-Baptiste Muselemu

**Affiliations:** 1 RAISE Initiative, Heilbrunn Department of Population and Family Health, Mailman School of Public Health, Columbia University, New York, New York, United States of America; 2 Save the Children US, Washington, DC, United States of America; 3 CARE USA, Atlanta, Georgia, United States of America; 4 International Rescue Committee, Kinshasa, Democratic Republic of the Congo; 5 CARE, Goma, Democratic Republic of the Congo; 6 Save the Children, Goma, Democratic Republic of the Congo; Johns Hopkins Bloomberg School of Public Health, UNITED STATES

## Abstract

**Context:**

Over 20 years of conflict in the DRC, North and South Kivu have experienced cycles of stability and conflict, resulting in a compromised health system and poor sexual and reproductive health outcomes. Modern contraceptive use is low (7.5%) and maternal mortality is high (846 deaths per 100,000 live births). Program partners have supported the Ministry of Health (MOH) in North and South Kivu to provide good quality contraceptive services in public health facilities since 2011.

**Methods:**

Cross-sectional population-based surveys were conducted in the program areas using a two-stage cluster sampling design to ensure representation in each of six rural health zones. Using MOH population estimates for villages in the catchment areas of supported health facilities, 25 clusters in each zone were selected using probability proportional to size. Within each cluster, 22 households were systematically selected, and one woman of reproductive age (15–49 years) was randomly selected from all eligible women in each household.

**Results:**

Modern contraceptive prevalence among women in union ranged from 8.4% to 26.7% in the six health zones; current use of long-acting or permanent method (LAPM) ranged from 2.5% to 19.8%. The majority of women (58.9% to 90.2%) reported receiving their current method for the first time at a health facility supported by the program partners. Over half of women in four health zones reported wanting to continue their method for five years or longer.

**Conclusion:**

Current modern contraceptive use and LAPM use were high in these six health zones compared to DRC Demographic and Health Survey data nationally and provincially. These results were accomplished across all six health zones despite their varied socio-demographic characteristics and different experiences of conflict and displacement. These findings demonstrate that women in these conflict-affected areas want contraception and will choose to use it when good quality services are available to them.

## Introduction

Globally, more than 65 million people were forcibly displaced from their homes at the end of 2016, the highest number since World War II; 40 million of these were displaced within their own countries [1,2]. Although acute crises dominate current headlines, people have been displaced for 10 years or longer in nearly 90% of the 60 countries monitored by the Internal Displacement Monitoring Centre [1]. Many have been displaced multiple times by chronic and recurring conflict. Armed conflict leads to complex humanitarian emergencies which are typically characterized by population displacement, the degradation of national health systems, and disruption of social order and norms [3,4]. Women are often disproportionately affected by humanitarian crises, for in addition to their need for food, water, shelter, and primary health care, they face a multitude of sexual and reproductive health (SRH) challenges including higher risks of morbidity and mortality due to pregnancy-related causes, mistimed pregnancy due to lack of information or availability of contraceptive services, complications resulting from unsafe abortion, gender-based violence and sexually transmitted infections including HIV [5].

Few of these women, however, have access to comprehensive SRH services.

An Inter-Agency Working Group on Reproductive Health in Crises global evaluation in 2012–2014 identified significant gaps with respect to contraceptive services available in humanitarian settings, with long-acting reversible contraception (LARC) rarely available [6–8]. While pre-existing awareness and use of contraception is also influential, demand for spacing or limiting births is present, as in any population. For example, studies in six conflict-affected areas of Sudan, Uganda and the Democratic Republic of the Congo (DRC) found that 43% to 71% of women wanted to delay their next pregnancy or did not want any more children, but fewer than 20% of women were using a modern contraceptive method (and fewer than 3% in four of the six settings) [9]. This discrepancy between women’s fertility desires and contraceptive use is likely due to the lack of services: zero to just over one-third of assessed health facilities in the six settings had the necessary staff and supplies to provide mandated contraceptive services. The repositioning of contraception in the 2018 revision of the Interagency field manual on reproductive health in humanitarian settings recognizes the critical importance of contraception in reducing maternal mortality [10].

Meeting unmet need for contraception could potentially prevent 30% of maternal deaths as well as contribute to improvements in child survival and reductions in poverty and hunger [11,12]. A broad range of contraceptive methods is essential to quality contraceptive programming in order to respond to individual preferences and changes over the course of a woman’s life [13,14]. Increasing contraceptive method choice is associated with increases in contraceptive prevalence [15]. Therefore, provision of a broad range of contraceptive methods is important. Evidence shows that a variety of approaches, including demand-side and supply-side contraceptive services interventions, have been successful at improving knowledge, attitudes and intentions to use contraception [16].

### Context and program description

Over 20 years of conflict in the DRC has resulted in a debilitated health system and poor SRH outcomes. A surge in violence in DRC in 2017 caused 2.2 million people to flee their homes resulting in 4.4 million internally displaced people, 1.1 million of whom are in North Kivu [17]. North and South Kivu have experienced cycles of stability and conflict over the last two decades. Modern contraceptive use is low (7.5%) and maternal mortality is high (846 deaths per 100,000 live births) [18]. In a 2014 national survey of health facilities, only 33% offered contraceptive services, of which 20% provided good quality services [19]. Short-acting methods (male condoms, contraceptive pills and injectables) were the mostly commonly available methods. A 2013 assessment in Masisi, North Kivu found that less than half of 25 assessed health centers had the necessary supplies and trained staff to provide contraceptive methods, from 48% for oral contraceptives to 20% for implants [8]. Since 2012, the DRC government has shown stronger political support for contraception, including an ambitious goal of modern contraceptive prevalence of 19% by 2020; however, this commitment has advanced more slowly at the provincial level [20,21].

CARE, International Rescue Committee (IRC) and Save the Children, in collaboration with the Reproductive Health Access, Information and Services in Emergencies (RAISE) Initiative at Columbia University, have supported the Ministry of Health (MOH) in North and South Kivu provinces to provide good quality contraceptive services in MOH health centers and hospitals in six health zones since 2011. As documented previously, support included the essential elements of good quality services [22]: method choice, clinical competence and supportive supervision of providers, counselling skills including the information given to clients, interpersonal skills, support for continuation of method use and integration with other health services [23–26]. Contraceptive services were provided free of charge. The programs emphasized quality improvement. The improvement and maintenance of health worker skills and competence has been a key element of the support. The partners also engaged in values clarification and attitudes transformation activities with providers. When specific weaknesses were identified, such as provider bias or lack of confidence with IUD insertion skills, the programs made changes to address them. These included strategies such as improved training on counseling, supportive supervision and coaching of providers to improve competence. The partners employed a variety of community engagement strategies to improve contraceptive uptake, and expanded the range of community mobilizers and activities to educate the community about contraception. They established more systematic supervision and support of these community actors and regularly discussed challenges they faced and how to overcome them. Encouraging providers to understand and use their clinical data motivated them to identify and develop solutions to problems in their facilities. Finally, improvements to logistics systems ensured consistent availability of contraceptive commodities. Although the partners followed the same overall program model, each organization carried out their own program with some variation in how they implemented the above components. All three partners are active members of the national and provincial Family Planning Stakeholders Groups (Comité Technique Multisectoriel Permanent, CTMP).

Other research conducted by RAISE and Save the Children in two health zones found that the program attracted early adopters of contraception and demonstrated that these new acceptors could also be supported to continue contraceptive use as long as they wished [23]. Anecdotal evidence from facility registers showed that a significant minority of clients traveled from outside the facility catchment areas to obtain contraception suggesting that women had heard these services were available and were subsequently motivated to travel some distance to obtain them, often selecting LARCs, as found in stable settings [15,27].

In 2016–2017, these partners conducted a program evaluation in North and South Kivu to measure population-level contraceptive prevalence in program areas. This manuscript describes the contraceptive prevalence results from this evaluation.

## Methodology

### Study design and sample

Cross-sectional population-based surveys were conducted in the program areas in six health zones: Kayna, Lubero, Masisi and Mweso in North Kivu and Kabare and Kalehe in South Kivu. The surveys used a two-stage cluster sampling design to ensure representation in each health zone. Sampling was based on a 95% confidence interval and 50% contraceptive prevalence, the most conservative estimate which requires the largest sample size [28]. Facilities that the partners began supporting less than 12 months previously were excluded from the sampling frame due to their limited exposure to the program. Villages that were deemed insecure at the time of the fieldwork were excluded from the sampling frame; this was most relevant in Lubero, Mweso and Masisi. Table 1 indicates the total population of the catchment areas of the supported facilities and the total population included in the sampling frame. Using 2016 and 2017 MOH population estimates for villages in the catchment areas of each health facility supported by a program partner, 25 clusters in each zone were selected using probability proportional to size. Villages with less than 600 people were combined with the next nearest village before cluster selection. Due to the volatile security situation in Masisi and Mweso, an extra cluster was selected in each health zone for a total of 26 in case one cluster became inaccessible once fieldwork began. Anticipating a high response rate similar to that achieved by the authors in prior research in DRC, 23 households were systematically selected in each cluster to ensure a minimum sample size of 500 respondents. One woman of reproductive age (15–49 years) was randomly selected from all eligible women in each household (listed by the interviewer with an adult member of the household) using a Kish grid (a pre-assigned table of random numbers used to select respondents) [29]. In all sites, if no one was home at a selected household, the interviewer went to the next closest house. If the selected woman was not home, an appointment was arranged to meet with the interviewer later that same day. If the selected woman was unavailable that day, the house was replaced by the next closest house. Due to security limitations, the teams were able to spend only one day in each cluster. Once a woman agreed to participate, a trained female interviewer interviewed her in private.

**Table 1:** Population of supported facilities and included in sampling frame.

Province	Health zone	Supported health facilities	Total population in catchment areas of supported facilities	Total population of villages included in sampling frame*
North Kivu	Kayna	4 health facilities	74,369	71,558 (96%)
	Lubero	4 health facilities	104,732	68,646 (66%)
	Masisi	12 health facilities	188,865	146.248 (77%)
	Mweso	10 health facilities	167,341	90,628 (54%)
South Kivu	Kalehe	10 health facilities	127,123	127,123 (100%)
	Kabare	15 health facilities	192,110	192,110 (100%)

*Villages in the catchment areas of supported facilities were excluded if they were deemed insecure and dangerous for interviewers to visit at the time of the survey.

### Study procedures

The survey questionnaire was adapted from questionnaires of the *Demographic and Health Surveys*, Ipas and those previously used by RAISE. The questionnaire covered women’s knowledge, attitudes and behaviors regarding contraception, abortion and post-abortion care. The questionnaire was developed in French and then translated into Congolese Swahili for all six health zones plus Kinyarwanda for Masisi and Mweso. The translation was reviewed for accuracy and revised by the survey teams. The questionnaire was piloted in villages not included in the survey samples.

In Kayna, Lubero, Kabare and Kalehe, paper questionnaires were used. In Masisi and Mweso, the questionnaire was programmed onto tablets using KoboToolbox. Female interviewers recruited locally were trained in SRH terminology and survey techniques, and participated in practical exercises to assure mastery of material. Interviewers discussed the different terms used in French and local languages for each contraceptive method and were trained to ask the woman where her method was placed to assure correct identification of current method used. Twenty interviewers and four supervisors were selected from the trainees in both Kabare/Kalehe and Kayna/Lubero while eighteen interviewers and three supervisors were selected in Masisi/Mweso. Supervisors reviewed completed questionnaires in the field, and interviewers were asked to return to the respondent if clarification was needed. In Masisi and Mweso, supervisors uploaded the data each evening when feasible; the first author reviewed the data and discussed feedback over the phone with the supervisors each morning. The dates of data collection can be found in Table 2.

**Table 2:** Number of respondents and dates of data collection.

Province	Health zone	Number of respondents	Response rate	Data collection
North Kivu	Kayna	551	96%	29 July to 12 August 2016
	Lubero	549	95%	29 July to 10 August 2016
	Masisi	550	96%	20 July to 7 August 2017
	Mweso	542	94%	11 July to 28 July 2017
South Kivu	Kalehe	554	96%	26 July to 10 August 2016
	Kabare	525	91%	22 July to 5 August 2016

### Ethical considerations

Respondents were asked to give oral informed consent; names were not entered on survey materials to preserve anonymity. Parental consent for women aged 15–17 was waived as this study met the criteria for minimal risk. Ethical approvals for the survey were obtained from the Institutional Review Board of the Mailman School of Public Health, Columbia University and the Institutional Ethical Commission of the Catholic University of Bukavu in DRC.

### Statistical analysis

Data were entered into CSPro 6.0 or KoboToolbox and subsequently exported to PASW (SPSS) Version 24 for cleaning and analysis. Data were weighted according to the number of eligible women of reproductive age in the household. Chi-square (categorical variables) and ANOVA (means) tests were used to describe basic characteristics and compare them across health zones. The primary outcome measures were current use of modern contraceptives and current use of a long-acting or permanent method (LAPM).

Modern contraceptive methods were defined as tubal ligation, vasectomy IUDs, implants, injectables, oral contraceptive pills and male and female condoms. Long-acting methods were defined as IUD and implant; permanent methods as tubal ligation and vasectomy. Contraceptive knowledge was defined as spontaneous or prompted knowledge of any modern method. Current contraceptive use results are reported for all women and for women in union (married or cohabitating).

## Results

The number of respondents interviewed in each health zone can be seen in Table 2. The mean age ranged from 26.5 years in Lubero to 28.4 years in Mweso (Table 3). The population was largely Christian, with some majority Catholic and some majority Protestant zones plus others that were more mixed. Education level differed among the zones from over half reporting no formal education in Masisi (50.7%) and Mweso (52.2%) while nearly half in Kayna (48%) reported some secondary school or higher. Most women were in union (currently married or cohabitating) in each zone (58.2% - 79.4%), except for Lubero where a slight majority (53.8%) reported being unmarried. Mean age at first marriage ranged from 17.1 years in Kalehe to 19.2 in Lubero while age at first sexual intercourse ranged from 14.1 years in Kalehe to 15.4 in Masisi. The majority of households reported ownership of a mobile phone, but this ranged from 50.4% in Mweso to 78.8% in Kayna. Experiences of displacement differed as well with Masisi (56.1%) and Mweso (87.9%) reporting the highest levels of having been displaced at least once in the previous five years compared to 6.8% in Kabare and 16.1% in Lubero.

**Table 3:** Sociodemographic characteristics.

	*Kayna*, *NK* (N = 940, 551)^1^ %(n)	*Lubero*, *NK* (N = 919, 549)^1^ %(n)	*Masisi*, *NK* (N = 704, 550)^1^ %(n)	*Mweso*, *NK* (N = 697, 542)^1^ %(n)	*Kabare*, *SK* (N = 806, 525)^1^ %(n)	*Kalehe*, *SK* (N = 928, 554)^1^ %(n)	p-value
**Age, mean (SD), years**	27.2 (8.6)	26.5 (8.6)	27.5 (8.0)	28.4 (8.4)	27.1 (8.6)	27.3 (9.3)	p = .002
**Age**							p < .001
15–19 years	21.2% (98)	25.8% (118)	17.5% (85)	19.7% (85)	19.9% (81)	27.0% (123)	
20–24 years	24.4% (131)	24.5% (132)	22.4% (129)	17.4% (102)	27.8% (146)	18.1% (109)	
25–49 years	54.5% (322)	49.7% (299)	60.1% (336)	63.0% (355)	52.4% (298)	54.8% (322)	
**Religion**							p < .001
Protestant	41.1% (232)	17.3% (92)	56.0% (311)	56.1% (307)	20.9% (119)	61.1% (357)	
Catholic	49.8% (273)	75.6% (425)	9.7% (54)	10.4% (55)	76.3% (394)	20.5% (110)	
Adventist	5.0% (24)	3.5% (15)	27.3% (148)	26.9% (144)	0.0% (0)	5.6% (28)	
Other or no religion	4.1% (22)	3.6% (17)	7.0% (36)	6.6% (35)	2.7% (11)	12.8% (58)	
**Education level**							p < .001
No formal schooling	16.2% (94)	14.9% (86)	50.7% (285)	52.2% (293)	34.1% (194)	40.4% (243)	
Primary school	35.8% (207)	42.5% (250)	29.5% (161)	33.7% (180)	26.7% (151)	24.9% (142)	
Secondary school or higher	48.0% (249)	42.5% (211)	19.7% (104)	14.1% (69)	39.2% (177)	34.7% (167)	
**Household owned mobile telephone**	78.8% (416)	73.4% (393)	62.1% (338)	50.4% (265)	68.4% (346)	59.9% (315)	p < .001
**Displaced at least once in previous 5 years**	24.3% (126)	16.1% (87)	56.1% (311)	87.9% (474)	6.8% (35)	31.2% (166)	p < .001
**Displaced now**	14.0% (69)	6.5% (32)	23.3% (133)	47.1% (255)	1.6% (9)	16.9% (91)	p < .001
**Marital status**							p < .001
Married or cohabitating	58.2% (345)	46.2% (289)	79.4% (457)	74.0% (432)	70.3% (412)	68.0% (419)	
Not married or cohabitating	41.8% (204)	53.8% (259)	20.6% (93)	26.0% (110)	29.7% (113)	32.0 (135)	
**Mean age at first marriage (SD), years**	18.8 (3.6)	19.2 (2.7)	17.4 (2.2)	17.5 (2.3)	19.0 (2.7)	17.1 (2.6)	p < .001
**Mean age at first sexual intercourse (SD), years**	15.1 (6.3)	14.4 (6.9)	15.4 (5.3)	15.0 (6.0)	14.7 (7.9)	14.1 (6.2)	p < .001

^1^N = weighted and unweighted base

Data are % of column weighted base (absolute counts), unless indicated. Bases are smaller for some variables due to missing data. Missing data are less than 0.5% for all variables.

Contraceptive knowledge was high with over 90% of respondents in all zones reporting knowledge of any modern contraceptive method, and a majority, ranging from 76.1% (Kabare) to 92.9% (Masisi), reported knowledge of any LAPM (Table 4). A health facility or friends and family members were the main sources of information about contraception, followed by community health workers (CHWs) and the radio. Husbands, included in the friends and family category, were a minimal source of information, reported by 0.8% to 6.0% of women.

**Table 4:** Contraceptive knowledge.

	*Kayna*, *NK* (N = 940, 551)^1^ %(n)	*Lubero*, *NK* (N = 919, 549)^1^ %(n)	*Masisi*, *NK* (N = 704, 550)^1^ %(n)	*Mweso*, *NK* (N = 697, 542)^1^ %(n)	*Kabare*, *SK* (N = 806, 525)^1^ %(n)	*Kalehe*, *SK* (N = 928, 554)^1^ %(n)	p-value
**Knowledge of any modern method**	94.7% (522)	91.5% (500)	97.6% (539)	97.7% (532)	92.3% (487)	94.2% (524)	p < .001
**Knowledge of any LAPM**	89.2% (498)	85.9% (471)	92.9% (516)	92.4% (504)	76.1% (422)	85.2% (474)	p < .001
**Where heard about contraception for the first time**							
Health facility	73.6% (422)	58.3% (334)	75.7% (435)	75.8% (418)	78.3% (429)	77.8% (433)	p < .001
CHW	41.5% (231)	20.9% (118)	31.7% (176)	43.5% (236)	26.9% (132)	23.6% (133)	p < .001
Radio	55.7% (302)	19.9% (110)	25.4% (131)	22.1% (122)	25.7% (128)	20.8% (95)	p < .001
Friend/family member/husband	54.9% (309)	66.2% (354)	42.5% (225)	49.2% (267)	37.8% (188)	45.9% (228)	p < .001
Posters/brochures	1.4% (8)	1.1% (6)	6.4% (30)	2.0% (12)	12.0% (61)	4.0% (20)	p < .001
Church	7.6% (43)	5.4% (27)	0.9% (4)	1.1% (5)	7.5% (39)	4.0% (20)	p < .001
Never heard of contraception	0.0% (0)	0.0% (0)	1.6% (8)	2.4% (11)	4.6% (23)	2.5% (13)	p < .001
Other (e.g., NGO, school, community leader)	7.2% (32)	9.4% (37)	5.3% (27)	3.6% (16)	6.9% (27)	9.3% (52)	p < .001

^1^N = weighted and unweighted base

Data are % of column weighted base (absolute counts), unless indicated. Bases are smaller for some variables due to missing data. Missing data are less than 0.5% for all variables.

**Fig 1:**
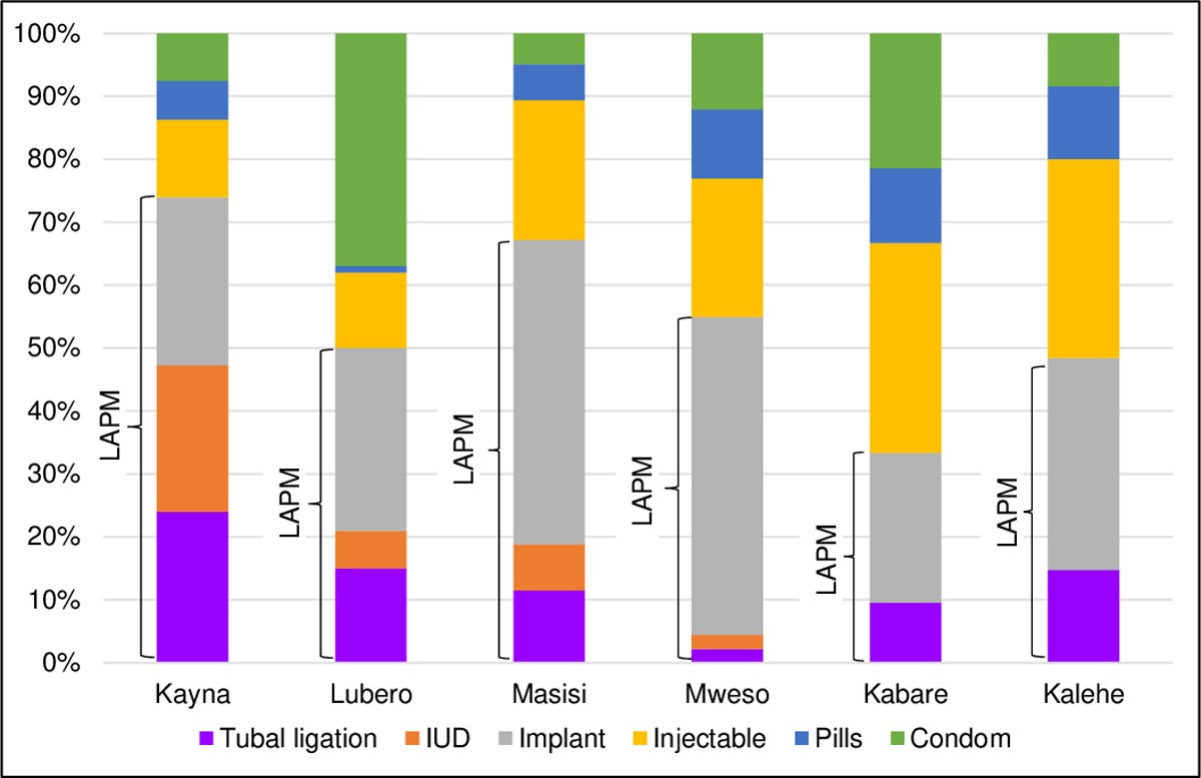
Method mix among women in union currently using modern contraception, North and South Kivu, DRC 2016 and 2017.

Modern contraceptive prevalence among women in union ranged from 8.4% in Kabare to 26.7% in Kayna; current use of an LAPM ranged from 2.5% in Kabare to 19.8% in Kayna (Table 5). Ever use of modern contraceptives and LAPM was also high ranging from 20.8% and 8.5% respectively in Kabare to 26.0% and 44.4% in Kayna. Implants were the most commonly used method among women in union (23.8% to 50.5%) except in Lubero where condoms (37.0%) were the most used (Fig 1 and S1 Table). Implants were the most popular method among all age groups in four of the six health zones; condoms were most popular across age groups in Lubero and among the two younger groups in Kabare (S1 Table). The majority of women reported receiving their current method for the first time at a health facility supported by the partners, from 58.9% in Lubero to 90.2% in Kalehe. Of those who did not receive their method at a supported health facility, over 50% used condoms or pills. More than 25% of current users reported using their current method for two years or more in 4 health zones (16.8%-40.7%). The majority of women reported their husband or partner was aware of her contraceptive use (76.3%-90.0%). While most women also reported that the decision to use a method was a joint one made with her husband or partner, this ranged from just over half (55.0%) in Kabare and Mweso to 83.3% in Kayna. From 14.4% to 39.3% of current method users reported having had at least one problem with their method: primarily changes in the menstrual period, followed by other side effects. However, nearly all reported current satisfaction with their method (89.8%-99.3%) and planned to continue using the method (72.8%-95.6%). Over half of women in four health zones reported wanting to continue their method for 5 years or longer.

**Table 5:** Current use of modern contraceptive methods.

	*Kayna*, *NK* (N = 940, 551)^1^ %(n)	*Lubero*, *NK* (N = 919, 549)^1^ %(n)	*Masisi*, *NK* (N = 704, 550)^1^ %(n)	*Mweso*, *NK* (N = 697, 542)^1^ %(n)	*Kabare*, *SK* (N = 806, 525)^1^ %(n)	*Kalehe*, *SK* (N = 928, 554)^1^ %(n)	p-value
**Reported ever use**^2^							
Modern method	44.4% (213)	42.1% (188)	44.0% (217)	43.3% (209)	20.8% (86)	29.6% (142)	p < .001
LAPM	26.0% (125)	16.7% (81)	25.5% (124)	23.0% (112)	8.5% (39)	11.5% (54)	p < .001
**Current modern contraceptive use**^**3**^							
*All women*							
Modern method	21.8% (117)	20.2% (113)	18.8% (100)	18.2% (98)	12.9% (66)	7.1% (33)	p < .001
LAPM	16.1% (83)	7.9% (51)	12.8% (64)	11.0% (58)	5.6% (27)	3.1% (16)	p < .001
*Women in union*							
Modern method	26.7% (83)	23.6% (66)	21.8% (91)	17.6% (77)	7.4% (27)	15.1% (54)	p < .001
LAPM	19.8% (58)	11.8% (35)	14.7% (57)	9.7% (42)	2.5% (11)	7.3% (25)	p < .001
**First source of current modern method**							p < .001
Supported health facility	69.8% (84)	58.9% (71)	78.8% (77)	80.2% (78)	62.5% (22)	90.2% (56)	
Non-supported health facility	22.9% (24)	11.1% (11)	16.7% (19)	16.7% (16)	5.4% (3)	5.4% (5)	
Pharmacy or boutique	5.9% (8)	23.9% (24)	3.8% (3)	1.6% (2)	17.9% (4)	4.5% (3)	
Other	1.5% (1)	6.1% (4)	1.6% (1)	1.6% (1)	14.3% (3)	0% (0)	
**Duration of current method use**							p < .001
Less than 12 months	49.3% (66)	44.6% (56)	54.8% (56)	48.9% (53)	48.9% (28)	28.9% (29)	
12–23 months	22.7% (31)	24.9% (25)	26.7% (28)	34.3% (35)	22.8% (12)	30.4% (21)	
2 years or more	27.9% (36)	30.6% (38)	18.5% (19)	16.8% (19)	28.3% (16)	40.7% (26)	
**Husband is aware of her contraceptive use**	90.0% (114)	87.8% (97)	80.6% (81)	78.3% (82)	76.3% (44)	77.7% (58)	p = .003
**Joint decision to use contraception**	83.3% (106)	79.3% (84)	68.7% (70)	55.0% (59)	55.0% (34)	71.7% (53)	p < .001
**Had at least one problem with current method**	25.3% (31)	14.4% (19)	39.3% (40)	33.1% (34)	15.2% (10)	22.4% (18)	p < .001
**Plans to continue method use**	91.7% (118)	92.9% (96)	95.6% (98)	94.1% (99)	72.8% (41)	94.6% (71)	p < .001
**Satisfaction with method**	93.4% (124)	89.8% (102)	97.7% (99)	99.3% (104)	87.0% (46)	91.8% (69)	p = .001
**Planned duration of continued method use**							
Less than 2 years	11.2% (13)	2.7% (2)	6.2% (5)	5.5% (6)	5.9% (4)	4.9% (5)	p < .001
2–4 years	23.4% (30)	29.3% (29)	41.9% (45)	56.3% (54)	22.1% (9)	34.4% (23)	
5 years or more or until menopause	59.0% (64)	58.0% (53)	51.9% (48)	28.3% (39)	39.7% (20)	53.3% (38)	
Not sure	6.3% (7)	10% (8)	0.0% (0)	0.0% (0)	32.4% (9)	7.4% (5)	

^1^N = weighted and unweighted base

^2^Women who have never had sexual intercourse are excluded.

^3^Modern contraceptive methods include tubal ligation, IUDs, implants, oral contraceptive pills, injectables and condoms. Long-acting or permanent methods (LAPM) include tubal ligation, IUDs and implants. No clients reported their partner had a vasectomy.

Data are % of column weighted base (absolute counts), unless indicated. Bases are smaller for some variables due to missing data. Missing data are less than 0.5% for all variables.

Among the women who were not currently using a modern contraceptive, the most commonly cited reasons (42.5%-62.0%) were those related to fertility, including a desire to get pregnant or infrequent sexual activity (Table 6). Approximately one-third of women in all health zones reported method-related reasons, primarily side effects (24.0%-37.0%). Opposition to contraceptive use (by the woman, her husband or family or her religion) was cited by 25.8% of women in Lubero to 34.3% in Kayna; 14.4%-24.0% reported their husband was opposed to contraception.

**Table 6:** Barriers to contraceptive use reported by women not currently using any contraception.

	*Kayna*, *NK* (N = 940, 551)^1^ %(n)	*Lubero*, *NK* (N = 919, 549)^1^ %(n)	*Masisi*, *NK* (N = 704, 550)^1^ %(n)	*Mweso*, *NK* (N = 697, 542)^1^ %(n)	*Kabare*, *SK* (N = 806, 525)^1^ %(n)	*Kalehe*, *SK* (N = 928, 554)^1^ %(n)	p-value
**Barriers to contraceptive use**							
Fertility-related reasons^2^	62.0% (234)	52.5% (197)	43.8% (188)	42.5% (168)	50.8% (210)	61.9% (277)	p < .001
Opposition to use^3^	25.8% (117)	36.7% (170)	29.2% (133)	30.9% (134)	34.1% (175)	34.2% (189)	p < .001
Lack of knowledge^4^	9.2% (36)	8.0% (32)	6.7% (27)	10.4% (47)	9.7% (43)	5.5% (26)	p = .006
Method-related reasons^5^	36.7% (172)	37.0% (164)	32.5% (142)	32.3% (148)	32.7% (167)	24.0% (126)	p < .001
Lack of access^6^	1.4% (6)	2.6% (11)	3.2% (13)	1.8% (8)	0.6% (3)	1.8% (9)	p = .012
Other^7^	0.3% (1)	0.6% (4)	6.0% (29)	5.2% (26)	5.7% (30)	4.3% (23)	p < .001
**Among women who went to a health facility in the last 12 months, provider discussed contraception**	55.4% (160)	32.7% (69)	57.6% (191)	46.9% (153)	46.7% (114)	56.5% (143)	p < .001

^1^N = weighted and unweighted base

^2^*Fertility-related reasons* include those who want to become pregnant or who are currently pregnant; are not married or whose husband is absent; are not having sex or infrequent sex; are (or her partner is) unable or having difficulty getting pregnant; are in menopause or had a hysterectomy; are postpartum or breastfeeding.

^3^*Opposition to use* includes those who oppose contraceptive use or don’t want to use contraception; whose husband opposes or others oppose contraceptive use; report religious prohibition; heard or believe that contraception is bad for her.

^4^*Lack of knowledge* includes those who know no method; know no source of methods; lack information or don’t have enough information about contraception; or say they have never heard of contraception.

^5^*Method-related reasons* include those who fear side effects; say that the method is inconvenient or difficult to use; report health-related reasons or say that contraception doesn’t work.

^6^*Lack of access* includes those who say services are too far; her preferred method is not available; it’s too expensive; the services are not confidential; the providers have bad attitudes.

^7^*Other* includes those who want to wait for a particular number of births before using; said she is not yet ready to use; or that she doesn’t need contraception.

Data are % of column weighted base (absolute counts), unless indicated. Bases are smaller for some variables due to missing data. Missing data are less than 0.5% for all variables.

## Discussion

**Fig 2:**
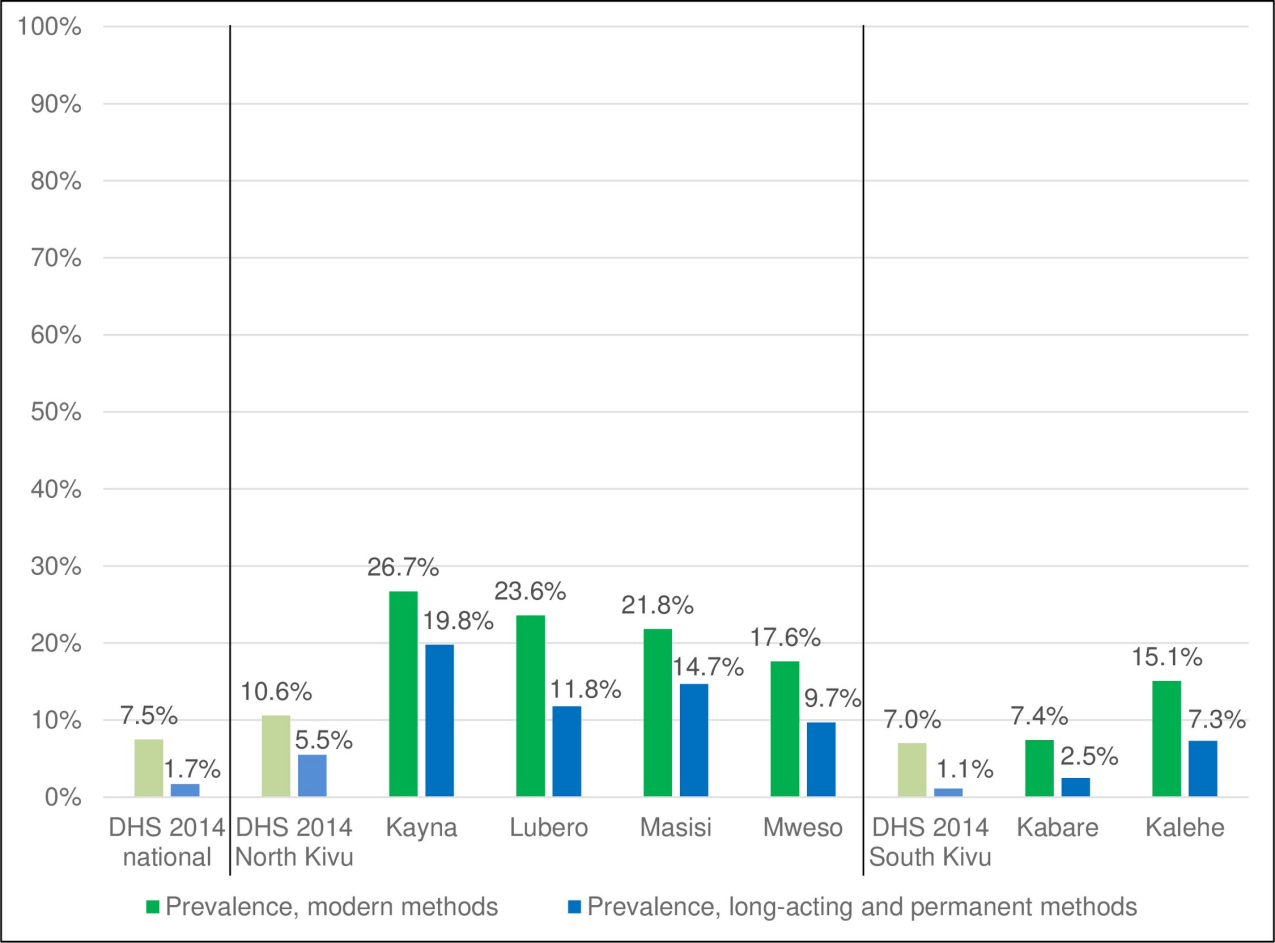
Contraceptive prevalence among women in union, modern and long-acting or permanent methods, North and South Kivu, DRC 2016 and 2017. Modern contraceptive methods include tubal ligation, IUDs, implants, injectables, oral contraceptive pills and condoms. Long-acting and permanent methods include as IUDs, implants and tubal ligation.

Current modern contraceptive use and LAPM use were high in these six health zones compared to DRC Demographic and Health Survey (DHS) data both nationally and provincially, likely due to the availability of good quality contraceptive services (Fig 2). These results compare favorably to other research conducted by the authors in these areas [23,24,30]. Most current users obtained their method from a supported health facility suggesting these results are likely due to our programs; over half of those who obtained their method elsewhere used condoms or pills. Implants were the most commonly used method followed by injectables except in Lubero where condoms were the most used and in Kayna where IUDs were the second most popular method. It is also important to note that LARC use was high across all age groups.

These results were accomplished across all six health zones despite their varied socio-demographic characteristics and different experiences of conflict and displacement. Use of modern contraception and of LAPM was high in the health zones most affected by the conflict and displacement (Masisi and Mweso) supporting the hypothesis that women affected by war and displacement want and will use contraception. Contraceptive use was high in health zones where half of women had no formal schooling (Masisi and Mweso) while also high in zones where nearly half of women had some secondary education (Lubero and Kayna). These services have the potential to bring about social change by enabling women, many for the first time, to plan when and if they became pregnant.

Contraceptive use in South Kivu was generally lower than that found in North Kivu, which is also consistent with DHS data [18]. It is unclear why Kabare health zone had the lowest contraceptive prevalence. Program staff believe this is likely due to active opposition to contraception by some priests at the Catholic mission in Kabare, including periodic calls from the pulpit to excommunicate women in the community who used implants and IUDs. A study in Malawi found that an individual religious leader’s beliefs about contraception is strongly associated with the contraceptive behavior of women in that congregation [31]. It is also possible that some women in Kabare may have chosen not to disclose their contraceptive use given this stigmatizing environment. It is, however, important to note that Lubero, which has a similarly high percentage of Catholics in the community, had higher contraceptive use, similar to that found in the other zones. This is consistent with studies showing similar contraceptive use among Catholics and women of other denominations [31,32].

While over 75% of women in each zone reported that her husband or partner was aware of her contraceptive use, lower percentages reported that the decision to use contraception was made jointly. For example, just over half (55%) of contraceptive users in Kabare and Mweso reported making the decision to use jointly their husband or partner. This could be explained by the stigma described above in Kabare and perhaps by the lower education levels among women in Mweso. In DRC, husbands are seen as having influence over the decision to use contraception, and are considered to have more agency than the woman in making this decision [33,34]. Among non-users who reported opposition to contraception as a barrier, women were more likely to report their husbands as being opposed than they themselves. This suggests a need for further outreach and education about the benefits of contraceptive use to men.

Less than one-third of women reported having a problem with their current method, and the vast majority of women planned to continue using their method. Satisfaction with their current method was very high in all health zones. These data suggest that efforts to support health workers to counsel on and treat side effects may be successful. Current users in South Kivu and Lubero where LAPM use was lower reported wanting to continue to use their method for a mean seven to eight years suggesting that more women may be interested in using a LAPM.

Fertility-related reasons were the most commonly cited reasons for non-use among women who were not currently using a modern contraceptive method. Approximately one-third of non-users reported method-related reasons as a barrier, consistent with studies in other countries [35,36]. Similar proportions of non-users reported opposition to contraceptive use as a barrier. This suggests a need to continue to discuss side effects and to dispel myths about methods in the community.

Women reported having heard about contraception for the first time primarily at health facilities. This suggests successful integration of contraception into health services, and that providers are providing good information. Friends and family members were the second most commonly cited source of information. It is likely that the majority of women living in these health zones know at least one woman who uses contraception. Husbands were cited as a source of information in very few cases, consistent with the opposition reported by non-users above. CHWs were also often cited as sources of information. These programs have worked closely with CHWs and satisfied users to discuss contraception and especially LARC, in the community.

It is important to note that these CARE, IRC and Save the Children programs worked through the MOH and supported MOH facilities and health workers thus strengthening the health system. Health system strengthening is a long-term process, but is an important component of post-conflict recovery [4]. The positive results found in these studies took time to achieve, suggesting a need for multi-year funding, but are more likely to be sustained as all program components were implemented in collaboration with the MOH. While dedicated donor funding was certainly critical to achieving such positive results, the capacity-strengthening of MOH staff should continue to contribute to improvements in contraceptive services in DRC, especially in light of the government’s recent commitments to contraceptive services [21]. In five of the six program areas, modern contraceptive prevalence equaled or surpassed the government’s goal of 19% prevalence by 2020. The partners have been active participants in the stakeholders groups (CTMP) which likely contributed to this achievement despite the challenges caused by the conflict and instability in these areas.

### Limitations

This evaluation lacks a baseline or control communities in the same areas which limits our ability to attribute the findings to our program efforts. However, the high numbers of current users reporting a program facility as the source of method help to infer our contribution. While our goal was to have representative samples of the program areas, in three health zones (Lubero, Masisi and Mweso), insecurity prevented access to some villages so the data are not representative of the full program area. It is likely that results would be lower in the most insecure areas. The survey was conducted one year later in Masisi and Mweso which may have affected responses. Service delivery continued through the time of the survey in these two zones, and they are located far from the other health zones so it is unlikely that there would have been contamination from doing the survey in the other zones first.

## Conclusion

These findings demonstrate that women in these conflict-affected areas want contraception and will choose to use it when good quality services are available to them. The support for health system strengthening and the strong focus on quality of care contributes to long-term recovery in a protracted crisis setting and sustainability; multi-year donor funding was crucial to these efforts. These results strengthen the evidence base for the implementation of contraceptive services in humanitarian settings, and demonstrate that effective programs resulting in adoption of contraceptive methods can be successfully implemented in these challenging settings.

## Supporting information

S1 Table Modern method mix by age group and marital status, by health zone, DRC 2016 and 2017. (DOCX)

S1 Survey Questions In English, French, Swahili and Kinyarwanda (from Kobo). (XLSX)
