# Supplementary material for: Meeting the demand of women affected by ongoing crisis: Increasing contraceptive prevalence in North and South Kivu, Democratic Republic of the Congo
Source: PLoS One. 2019 Jul 19;14(7):e0219990. doi: 10.1371/journal.pone.0219990 (PMC6641211; doi:10.1371/journal.pone.0219990)
Supplement: S1 Table — (DOCX) [file pone.0219990.s001.docx]

**S1 Table: Modern method mix by age group and marital status, by health zone, North and South Kivu, DRC, 2016 and 2017**

|  | **Any modern method** | **Tubal ligation** | **IUD** | **Implant** | **Injectable** | **Pills** | **Condom** | **p-value / *unweighted n*** |
| --- | --- | --- | --- | --- | --- | --- | --- | --- |
| **Kayna** |  |  |  |  |  |  |  |  |
| *By age* |  |  |  |  |  |  |  | p<.001 |
| 15-19 years | 13.1% | 0 | 38.5% | 23.1% | 11.5% | 11.5% | 15.4% | *n=13* |
| 20-24 years | 15.7% | 0 | 8.3% | 52.8% | 2.8% | 8.3% | 27.8% | *n=23* |
| 25-49 years | 27.9% | 24.5% | 23.8% | 30.8% | 11.9% | 3.5% | 5.6% | *n=81* |
| *By marital status* |  |  |  |  |  |  |  | p<.001 |
| Married or cohabitating | 26.7% | 24.0% | 23.3% | 26.7% | 12.3% | 6.2% | 7.5% | *n=83* |
| Unmarried | 14.8% | 0 | 22.4% | 51.7% | 3.4% | 3.4% | 19.0% | *n=33* |
| **Lubero** |  |  |  |  |  |  |  |  |
| *By age* |  |  |  |  |  |  |  | p=.003 |
| 15-19 years | 9.3% | 0 | 9.1% | 22.7% | 13.6% | 0 | 54.5% | *n=12* |
| 20-24 years | 25.8% | 3.4% | 6.9% | 13.8% | 1.7% | 6.9% | 67.2% | *n=27* |
| 25-49 years | 23.2% | 15.1% | 5.7% | 28.3% | 11.3% | 1.9% | 37.7% | *n=74* |
| *By marital status* |  |  |  |  |  |  |  | p<.001 |
| Married or cohabitating | 23.6% | 15.0% | 6.0% | 29.0% | 12.0% | 1.0% | 37.0% | *n=66* |
| Unmarried | 17.4% | 3.5% | 7.0% | 16.3% | 4.7% | 5.8% | 62.8% | *n=47* |
| **Masisi** |  |  |  |  |  |  |  |  |
| *By age* |  |  |  |  |  |  |  | p=.028 |
| 15-19 years | 8.1% | 0 | 0 | 90.0% | 0 | 0 | 10.0% | *n=8* |
| 20-24 years | 15.2% | 0 | 8.3% | 41.7% | 25.0% | 12.5% | 12.5% | *n=19* |
| 25-49 years | 23.2% | 14.3% | 8.2% | 48.0% | 23.5% | 4.1% | 2.0% | *n=73* |
| *By marital status* |  |  |  |  |  |  |  | p=.672 |
| Married or cohabitating | 21.8% | 11.5% | 7.4% | 48.4% | 22.1% | 5.7% | 4.9% | *n=91* |
| Unmarried | 6.9% | 0 | 10.0% | 70.0% | 20.0% | 0 | 0 | *n=9* |
| **Mweso** |  |  |  |  |  |  |  |  |
| *By age* |  |  |  |  |  |  |  | p=.147 |
| 15-19 years | 8.8% | 0 | 0 | 83.3% | 0 | 16.7% | 0 | *n=7* |
| 20-24 years | 20.7% | 0 | 0 | 52.0% | 36.0% | 12.0% | 0 | *n=20* |
| 25-49 years | 20.5% | 3.3% | 2.2% | 54.4% | 17.8% | 10.0% | 12.2% | *n=71* |
| *By marital status* |  |  |  |  |  |  |  | p=.147 |
| Married or cohabitating | 17.6% | 2.2% | 2.2% | 50.5% | 22.0% | 11.0% | 12.1% | *n=77* |
| Unmarried | 19.9% | 2.8% | 0 | 72.2% | 13.9% | 11.1% | 0 | *n=21* |
| **Kabare** |  |  |  |  |  |  |  |  |
| *By age* |  |  |  |  |  |  |  | p<.001 |
| 15-19 years | 5.0% | 0 | 0 | 0 | 0 | 37.5% | 62.5% | *n=3* |
| 20-24 years | 2.7% | 0 | 0 | 0 | 0 | 66.7% | 33.3% | *n=3* |
| 25-49 years | 10.2% | 9.3% | 0 | 48.8% | 32.6% | 4.7% | 4.7% | *n=27* |
| *By marital status* |  |  |  |  |  |  |  | p=.001 |
| Married or cohabitating | 7.4% | 9.5% | 0 | 23.8% | 33.3% | 11.9% | 21.4% | *n=27* |
| Unmarried | 6.3% | 0 | 0 | 73.3% | 0 | 26.7% | 0 | *n=6* |
| **Kalehe** |  |  |  |  |  |  |  |  |
| *By age* |  |  |  |  |  |  |  | p=.002 |
| 15-19 years | 3.6% | 0 |  | 33.3% | 33.3% | 0 | 33.3% | *n=5* |
| 20-24 years | 12.5% | 0 | 0 | 4.8% | 61.9% | 19.0% | 14.3% | *n=12* |
| 25-49 years | 17.7% | 15.6% | 0 | 37.8% | 32.2% | 7.8% | 6.7% | *n=49* |
| *By marital status* |  |  |  |  |  |  |  | p=.014 |
| Married or cohabitating | 15.1% | 14.7% | 0 | 33.% | 31.6% | 11.6% | 8.4% | *n=54* |
| Unmarried | 8.4% | 0 | 0 | 24.0% | 60.0% | 0 | 16.0% | *n=12* |
